# AXL regulates neuregulin1 expression leading to cetuximab resistance in head and neck cancer

**DOI:** 10.1186/s12885-022-09511-6

**Published:** 2022-04-23

**Authors:** Mari Iida, Nellie K. McDaniel, Kourtney L. Kostecki, Noah B. Welke, Carlene A. Kranjac, Peng Liu, Colin Longhurst, Justine Y. Bruce, Seungpyo Hong, Ravi Salgia, Deric L. Wheeler

**Affiliations:** 1 Department of Human Oncology, School of Medicine and Public Health, University of Wisconsin, 1111 highland Ave, WIMR 3159, Madison, WI 53705 USA; 2 School of Medicine and Public Health, University of Wisconsin Carbone Cancer Center, University of Wisconsin, Madison, WI USA; 3 Department of Biostatistics and Medical Informatics, School of Medicine and Public Health, University of Wisconsin, Madison, WI USA; 4 Department of Medicine, School of Medicine and Public Health, University of Wisconsin, Madison, WI USA; 5 Pharmaceutical Sciences Division, University of Wisconsin School of Pharmacy, Madison, WI USA; 6 Wisconsin Center for NanoBioSystems, University of Wisconsin, Madison, WI USA; 7 Yonsei Frontier Lab, Department of Pharmacy, Yonsei University, Seoul, Korea; 8 Department of Medical Oncology and Experimental Therapeutics, Comprehensive Cancer Center, City of Hope, Duarte, CA USA

**Keywords:** AXL, neuregulin1, Head and neck cancer, Cetuximab, Resistance, HER3

## Abstract

**Background:**

The receptor tyrosine kinase (RTK) epidermal growth factor receptor (EGFR) is overexpressed and an important therapeutic target in Head and Neck cancer (HNC). Cetuximab is currently the only EGFR-targeting agent approved by the FDA for treatment of HNC; however, intrinsic and acquired resistance to cetuximab is a major problem in the clinic. Our lab previously reported that AXL leads to cetuximab resistance via activation of HER3. In this study, we investigate the connection between AXL, HER3, and neuregulin1 (NRG1) gene expression with a focus on understanding how their interdependent signaling promotes resistance to cetuximab in HNC.

**Methods:**

Plasmid or siRNA transfections and cell-based assays were conducted to test cetuximab sensitivity. Quantitative PCR and immunoblot analysis were used to analyze gene and protein expression levels. Seven HNC patient-derived xenografts (PDXs) were evaluated for protein expression levels.

**Results:**

We found that HER3 expression was necessary but not sufficient for cetuximab resistance without AXL expression. Our results demonstrated that addition of the HER3 ligand NRG1 to cetuximab-sensitive HNC cells leads to cetuximab resistance. Further, AXL-overexpressing cells regulate NRG1 at the level of transcription, thereby promoting cetuximab resistance. Immunoblot analysis revealed that NRG1 expression was relatively high in cetuximab-resistant HNC PDXs compared to cetuximab-sensitive HNC PDXs. Finally, genetic inhibition of NRG1 resensitized AXL-overexpressing cells to cetuximab.

**Conclusions:**

The results of this study indicate that AXL may signal through HER3 via NRG1 to promote cetuximab resistance and that targeting of NRG1 could have significant clinical implications for HNC therapeutic approaches.

**Supplementary Information:**

The online version contains supplementary material available at 10.1186/s12885-022-09511-6.

## Background

Head and neck squamous cell carcinomas (HNSCC) develop from the mucosal lining of the aerodigestive tract. It is estimated that in 2020 there will be over 53,000 new cases of HNSCC in the United States and 11,000 deaths from this disease [1, 2]. HNSCC is a complex heterogeneous disease that arises from various sites including the oral cavity, tongue, pharynx, larynx, and salivary glands. Over 90% of tumors that originate in the oropharyngolaryngeal axis are squamous cell carcinomas. The 5-year relative survival rate for oral cavity and pharynx during 2009–2015 was 65% in the US [2]. The therapy regimen used to treat this cancer typically involves surgery, radiotherapy, chemotherapy, targeted therapy, and/or immunotherapy [3].

Common systemic agents that are utilized for treatment of HNC patients are monotherapy or combination regimens containing platinums, taxanes, immune checkpoint inhibitors, and cetuximab [4, 5]. Cetuximab is a chimeric monoclonal antibody targeting the epidermal growth factor receptor (EGFR) that was approved by the FDA for treatment of HNC in 2006. EGFR is a member of the HER family of receptor tyrosine kinases (RTKs), and aberrant expression of EGFR has been strongly noted in the etiology of HNSCC [3, 6]. Cetuximab is the only drug that exhibits efficacy in both locally advanced and recurrent/metastatic HNC. It functions by binding the extracellular domain of EGFR with high affinity, blocking ligand binding and stimulating receptor internalization. As an IgG1 class antibody, it also stimulates antibody dependent cell cytotoxicity [7]. Targeting of EGFR with cetuximab has improved overall survival of HNSCC patients when added to radiotherapy or chemotherapy regimens [8, 9]. Despite the clinical benefit of cetuximab treatment, resistance to cetuximab can develop in patients [10]. Thus, investigation of resistant mechanisms can provide new insights to improving treatment strategies in HNSCC.

The RTK AXL is a member of the TAM family of receptors (Tyro, AXL, MER) and has been implicated in the development and progression of many malignancies [11–14]. These studies indicate a role for AXL in cancer cell proliferation, migration, angiogenesis, and metastasis [14–16]. AXL mRNA expression has been correlated with poor disease outcome in HNSCC, indicating a putative role for AXL in the development and/or progression of this disease [17]. Moreover, AXL protein expression levels increased during HNSCC tumor progression [18]. Our laboratory previously reported that AXL expression was significantly associated with higher pathologic grade, distant metastases, and shorter relapse-free survival in HNSCC patients. Many studies have found that AXL can also mediate resistance to anti-EGFR inhibitors which further unveils a role for AXL in therapeutic resistance [11, 13, 19–24].

The RTK HER3 has been shown to be upregulated in many cancer types including HNSCC and has been correlated with invasion and metastasis [25–27]. It has also been reported that membranous HER3 expression is significantly associated with worse overall survival in HNSCC [28, 29]. Our laboratory reported that acquired resistance to cetuximab is accompanied by EGFR-dependent activation of HER3 in non-small cell lung cancer (NSCLC) and HNSCC [19, 30, 31]. We also revealed that genetic silencing of HER3 was able to resensitize cetuximab-resistant clones to cetuximab therapy, suggesting a role for HER3 in driving resistance [32]. This role is also established in a study that demonstrates inhibition of HER3 combined with cetuximab has strong anti-tumor activity in cetuximab-resistant HNSCC patient-derived xenografts [33]. All of these studies underscore the important role of HER3 in therapeutic resistance [26, 34]. Collectively, AXL or HER3 can play important roles in mediating resistance to cetuximab and that targeting of AXL or HER3 can resensitize to cetuximab treatment [31, 35].

Based on our previous studies of AXL and HER3 in the context of cetuximab resistance, we investigated the connection between these two receptors with a focus on understanding their interdependent signaling to promote resistance to cetuximab. A model of AXL overexpression revealed that AXL leads to cetuximab resistance via activation of HER3, but we found that genetic overexpression of HER3 is insufficient for cetuximab resistance. However, exogenous expression of the HER3 ligand neuregulin1 (NRG1) does lead to cetuximab resistance. Further experimentation revealed that AXL can regulate NRG1, at the level of mRNA, to promote cetuximab resistance. Other cetuximab-resistant models have relatively high AXL and NRG1 expression, and targeting of NRG1 in these models is sufficient to overcome cetuximab resistance. Collectively, this data indicates that AXL can signal through HER3 via NRG1 to promote cetuximab resistance and that targeting of NRG1 could have significant clinical implications for HNSCC therapeutic approaches.

## Materials and methods

### Reagents

Cetuximab (IMC-C225, Erbitux) was purchased from the University of Wisconsin Pharmacy. Gas6 and NRG1 were obtained from R&D systems (Minneapolis, MN). DMSO (MilliporeSigma, St. Louis, MO) was used as the vehicle control in vitro. Human IgG (MilliporeSigma, St. Louis, MO) was the control for cetuximab.

### Cell lines and HNSCC PDXs

UMSCC1, UMSCC6, and HNSCC PDXs were obtained from SPORE resources. The HN30 and PCI37A cell lines were a gift from Dr. Ravi Salgia, City of Hope, Duarte, CA and Dr. Jennifer Grandis, UCSF, CA, respectively. All cell lines were cultured in DMEM with 4.5 g/dL glucose, 10% FBS, penicillin (100 units/mL), and streptomycin (100 mg/mL). Cell line identity was confirmed using short tandem repeat analysis and publicly available databases by the TRIP lab at the University of Wisconsin-Madison. STR reference of PCI37A was not available in public, however the genomic integrity remains similar for more than 2 years. Mycoplasma testing was completed through the WiCell Core Service at the University of Wisconsin-Madison. Detailed information about cell lines and PDXs is listed in Supplemental Materials and Methods.

### Plasmids and transfection

Plasmids were prepared and selected as previously described [36]. Transfection was performed using Lipofectamine3000 and Opti-MEM (Life Technology, Carlsbad, CA) according to the manufacturer's instructions [31, 36]. Blasticidin (3ug/mL) was used as the selective antibiotic when maintaining cells.

### siRNA transfection

Non-targeting control pool siRNA (Cat#D-001810), SMARTpool siRNA targeting HER3 (Cat#L-003127), AXL (Cat#L-003104) and NRG1 (Cat#L-004608) were purchased from Dharmacon, Inc (Lafayette, CO) and utilized for transfection with Lipofectamine RNAiMAX (Life Technologies) [31]. Successful knockdown was confirmed by immunoblot.

### Cell-based assays

Cell Counting Kit-8 (CCK8, Dojindo Molecular Technologies, MD) and crystal violet assays were performed to determine cell proliferation or cell viability as described previously [37]. For the CCK8 assay, equal number of cells were plated on 96-well plates. Following treatment, CCK8 analysis was performed according to the manufacturer’s instructions. For the crystal violet assays, equal number of cells were seeded in 6-well plates. Following treatment, the cells were stained with 0.5% crystal violet. Plates were air dried, and dye was eluted with 0.1 M sodium citrate (pH 4.2) and ethanol (1:1). Elution was transferred to 96-well plates, and the absorbance was read at 540 nm to determine cell proliferation. For studies using combination treatment of siRNA and other agents, cells were first transfected with siRNA. Twenty-four hours later, cells were treated with the other agents for an additional 72 h. All treatments were performed in duplicate or triplicate.

### Immunoblot analysis

Whole-cell protein was obtained from cells using RIPA buffer (50 mM Hepes, pH 7.4, 150 mM NaCl, 0.1% Tween-20, 10% glycerol, 2.5 mM EGTA, 1 mM EDTA, 1 mM DTT, 1 mM Na3VO4, 1 mM PMSF, 1 mM beta-glycerophosphate (BGP), and 10 μg/ml leupeptin and aprotinin). Immunoblot analysis was performed as previously described [36, 37]. Antibodies were used according to the manufacturer’s instructions: AXL (Cell Signaling Technologies, MA (CST) #8661), pAXL/pMerTK/pTyro3 (CST #44463), HER3 (CST #12708), pHER3 Y1197 (CST #4561), AKT (CST #2920), pAKT (CST #4060), NRG1 (CST #2573), GAPDH (CST #2118), α-tubulin (MilliporeSigma #CP06).

### RNA isolation, cDNA synthesis and qPCR

RNA isolation, cDNA synthesis and qPCR were performed as previously described [31]. Briefly, total RNA was isolated from the cells using RNeasy kit (Qiagen). The RNA was then quantified by a NanoDrop spectrophotometer (Thermo Fisher Scientific, Waltham, MA). RNA was reverse transcribed to cDNA using qScript™ cDNA SuperMix (Quantabio, Beverly, MA). Quantitative real time qPCR was carried out using the Bio-Rad CFX96 real-time PCR system (Bio-Rad, Hercules, CA). Eukaryotic 18S rRNA (4,333,760, Life Technologies) and human ACTB (4,333,762, Life Technologies) were used as the normalization controls. The levels of gene expression were analyzed using the ΔΔC_t_ method.

### Statistical analysis

Statistical analyses were performed using Prism (GraphPad Software, Inc.). Differences between multiple groups were evaluated using a repeated measures ANOVA with a Bonferroni post-hoc test [38]. Differences were considered significant when *P* < 0.05.

## Results

### AXL leads to cetuximab resistance and increased HER3 activity

**Fig. 1:**
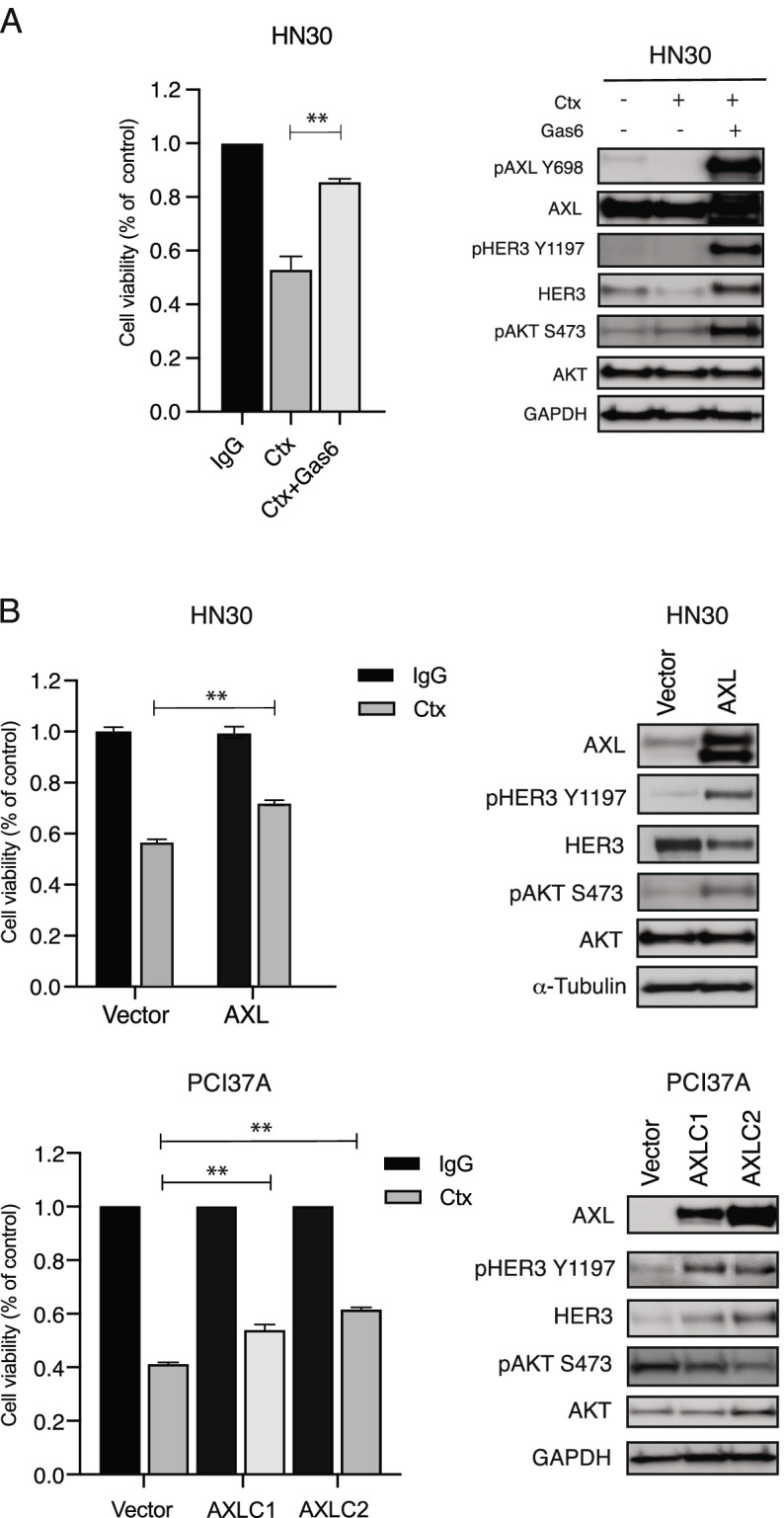
AXL leads to cetuximab resistance and increased HER3 activity. A: HN30 cells were treated with 100 nM of IgG, 100 nM of cetuximab (Ctx), or combination of Ctx and Gas6 (200 ng/uL) for 72 h, and cell viability was determined by crystal violet assay. Mean values, SEs, and statistical analyses are representative of two independent experiments. *N* = 3, **P < 0.01. Whole cell lysates were harvested and fractionated via SDS-PAGE, followed by immunoblotting for the indicated proteins. GAPDH was used as a loading control. B: Cell viability in AXL overexpressed cells was measured via crystal violet assay after 72 h of treatment with cetuximab. Mean values, SEs, and statistical analyses are representative of three independent experiments. *N* = 3, **P < 0.01. Whole cell lysates were harvested and fractionated via SDS-PAGE, followed by immunoblotting for the indicated proteins. α-Tubulin and GAPDH were used as loading controls

Previously, we reported that many cetuximab-resistant HNSCC cell lines exhibited increased expression and activation of AXL relative to cetuximab-sensitive cell lines. In addition, we showed that HNSCC cell lines that expressed AXL were dependent on this receptor for proliferation by using small interfering RNA (siRNA) analysis [35, 37]. Since siAXL impaired the proliferation of cetuximab-resistant HNSCC cells, we first sought to identify if ligand-induced activation of AXL may mediate cetuximab resistance. We stimulated cetuximab-sensitive HN30 cells with Gas6, an AXL ligand, to determine if this would lead to increased resistance to cetuximab. Stimulation of AXL by Gas6 in HN30 cells did result in resistance to cetuximab treatment (Fig. 1A). Immunoblot analysis indicated that HN30 cells stimulated with Gas6 had robust expression and phosphorylation of AXL and HER3 receptors, even in the presence of cetuximab. AKT was also highly phosphorylated by Gas6 stimulation. This result suggests that activation of AXL and HER3 by Gas6 can stimulate the cell proliferation and survival pathway, thereby escaping the inhibitory effects of cetuximab.

To further evaluate whether overexpression of AXL increased HER3 phosphorylation in HNSCC cells, we overexpressed AXL in the cetuximab-sensitive cell lines HN30 (HN30-AXL) and PCI37A (PCI37A-AXL) via stable transfection. Endogenous levels of AXL and HER3 in HN30 and PCI37A cell lines were evaluated via immunoblot (Supplemental Fig. 1). Proliferation assays demonstrated that AXL overexpressing cell lines were significantly resistant to increasing doses of cetuximab compared to vector cells (Fig. 1B). Immunoblot analysis confirmed that total AXL expression was increased in the cells, and it also indicated that phosphorylation of HER3 was increased in HN30-AXL and PCI37A-AXL cells. Collectively, these results suggest that overexpression of AXL leads to cetuximab resistance and increased HER3 activity.

### HER3 is necessary for AXL to mediate cetuximab resistance

**Fig. 2:**
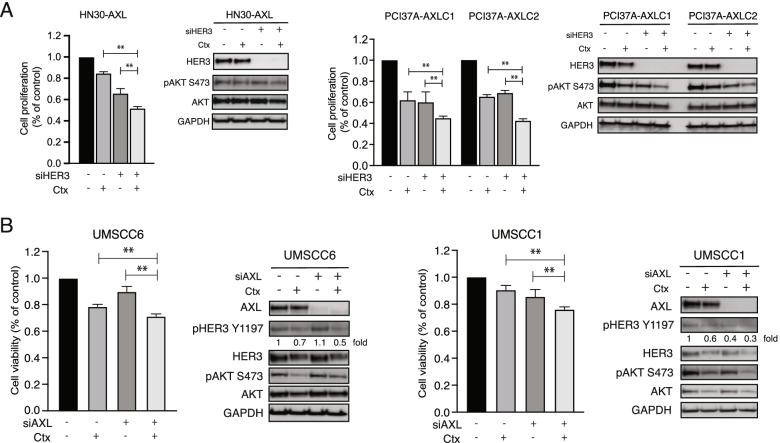
HER3 is necessary for AXL to mediate cetuximab resistance. **A** HN30-AXL and PCI37A-AXL cells were plated and treated with 30 nM of HER3 siRNA (siHER3) or 30 nM non-target siRNA (siNT). The next day, cells were treated with 100 nM of IgG or 100 nM cetuximab (Ctx) for 72 h. Cell proliferation was measured after drug treatment by CCK8 assay. Mean values, SEs, and statistical analyses are representative of three independent experiments. *N* = 5–10, **P < 0.01. Whole cell lysates were collected 24 h after treatment, fractionated by SDS-PAGE and immunoblotted for the indicated proteins. GAPDH was used as a loading control. **B** UMSCC1 and UMSCC6 HNSCC cells were plated and treated with 30 nM of siAXL or 30 nM siNT. The next day, cells were treated with 100 nM of IgG or 100 nM Ctx for 72 h. Cell viability was measured after drug treatment by crystal violet assay. Mean values, SEs, and statistical analyses are representative of three independent experiments. *N* = 3, **P < 0.01. Whole cell lysates were collected at 24 h after treatment, fractionated by SDS-PAGE, and immunoblotted for the indicated proteins. GAPDH was used as a loading control

To determine if HER3 is important in cetuximab-resistant cells whose resistance is caused by AXL overexpression, cell proliferation assays were performed using cetuximab treatment and siRNAs targeting HER3 (Fig. 2A). Cell proliferation of HN30-AXL, PCI37A-AXLC1, and PCI37A-AXLC2 cells was significantly inhibited when treated with the combination of siHER3 and cetuximab compared to either treatment alone. Immunoblot analysis showed that the combination of siHER3 and cetuximab treatment decreased phosphorylation of AKT.

**Fig. 3:**
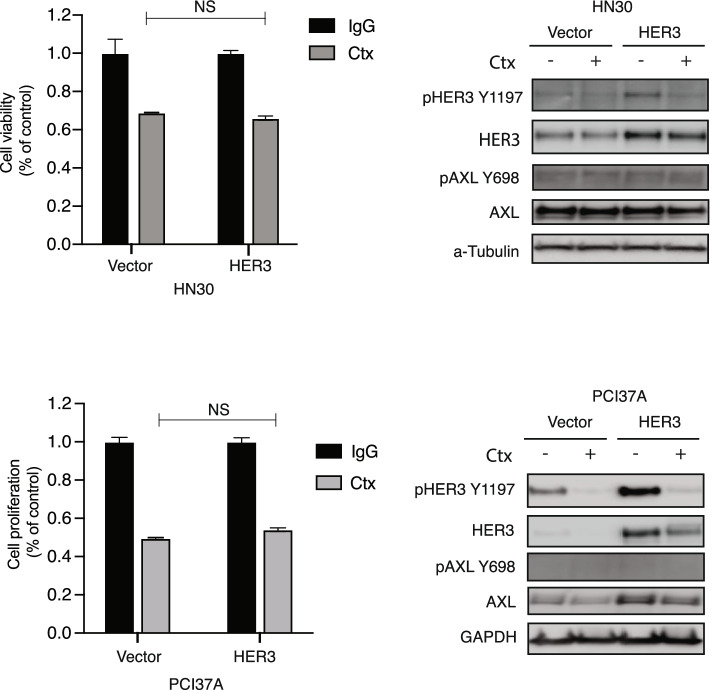
HER3 overexpression alone is insufficient for cetuximab resistance. A: HN30 and PCI37A cells stably overexpressing HER3 or the pcDNA6.0 vector were treated with 100 nM of cetuximab (Ctx) for 72 h before performing crystal violet assay for HN30 cells and CCK8 assays for PCI37A cells. Whole cell lysate was harvested at 24 h after treatment and subjected to immunoblot analysis following fractionation via SDS-PAGE. GAPDH or α-Tubulin was used as a loading control. Mean values, SEs, and statistical analyses are representative of two or three independent experiments. *N* = 3–6. NS: not significant

Because siHER3 inhibited the proliferation of HN30-AXL and PCI37A-AXL cells, we next investigated if targeting AXL in cell lines that are *intrinsically* resistant to cetuximab (UMSCC6, UMSCC1) would enhance cetuximab sensitivity and decrease phosphorylation of HER3. Cell proliferation analysis was performed after treatment of UMSCC6 and UMSCC1 HNSCC cells with sinontarget (siNT), cetuximab, siAXL, or the combination of siAXL and cetuximab. The results of these experiments demonstrated that the combination of siAXL and cetuximab had a significant anti-proliferative effect on these cells compared to either treatment alone (Fig. 2B). Immunoblot analysis indicated that phosphorylation of HER3 was inhibited by the combination treatment. These results indicate that HER3 signaling collaborates with AXL to regulate cellular proliferation and response to cetuximab. To further investigate whether the expression of HER3 alone is sufficient to mediate resistance to cetuximab, two HNSCC cell lines that are sensitive to cetuximab and express little or no endogenous HER3 were manipulated to overexpress HER3 and treated with cetuximab for cell proliferation analysis (Fig. 3). Results of this experiment indicated that both HN30 and PCI37A cells remained sensitive to cetuximab treatment even with overexpression of HER3. AXL phosphorylation levels were not increased in HN30-HER3 and PCI37A-HER3 cells. Collectively, these results demonstrate that HER3 overexpression without AXL activation is insufficient for cetuximab resistance.

### Exogenous expression of NRG1 leads to cetuximab resistance

**Fig. 4:**
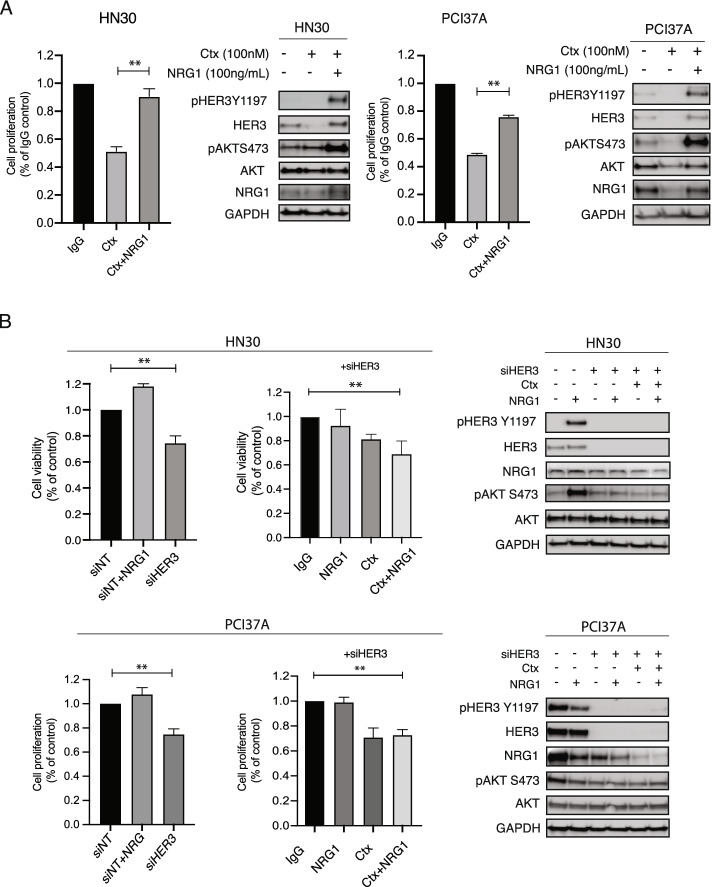
Exogenous expression of NRG1 leads to cetuximab resistance. **A** HN30 and PCI37A cells were plated and treated with 100 nM of cetuximab (Ctx), 100 ng/mL of NRG1, or the combination of Ctx and NRG1 for 72 h. Cell proliferation was determined by CCK8 assay. Mean values, SEs, and statistical analyses are representative of two independent experiments. *N* = 6, **P < 0.01. Whole cell lysates were harvested at 24 h after treatment and fractionated via SDS-PAGE, followed by immunoblotting for the indicated proteins. GAPDH was used as a loading control. **B** HN30 and PCI37A cells were transfected with 30 nM siHER3 or 30 nM siNT for 24 h before treatment with Ctx (100 nM) or NRG1 (100 ng/ml) for an additional 72 h. Cell viability or cell proliferation was determined by crystal violet assay for HN30 cells and CCK8 assay for PCI37A cells. Mean values, SEs, and statistical analyses are representative of two or three independent experiments. *N* = 3–10, **P < 0.01. Whole cell lysate was harvested at 24 h after treatment and subjected to immunoblot analysis following fractionation via SDS-PAGE. GAPDH was used as a loading control

We have previously reported that ligand-mediated activation of HER family receptors, especially HER3, could mediate resistance to cetuximab [31, 39]. In this study, however, HER3 expression was necessary but not sufficient for cetuximab resistance without AXL expression. Therefore, we hypothesized that another pathway downstream of AXL must influence expression of HER3 and the HER3 ligand, NRG1. To test this hypothesis, we first stimulated the HN30 and PCI37A cells with NRG1 to assess if this would lead to increased resistance to cetuximab. Addition of NRG1 to HN30 or PCI37A cells did result in resistance to cetuximab (Fig. 4A). Immunoblot analysis indicated that phosphorylation levels of HER3 and AKT were increased in both cell lines after NRG1 stimulation and not inhibited by cetuximab in the presence of NRG1. This result suggested that presence of NRG1 is sufficient to stimulate HER3, leading to regulation of cell proliferation and survival pathways, thus bypassing the inhibitory effects of cetuximab.

To determine the importance of HER3 activation in cetuximab resistance, we treated the cetuximab-sensitive cell lines HN30 and PCI37A with siHER3 and cetuximab for 72 h and subsequently stimulated them with exogenous NRG1. Analysis of cell proliferation indicated that both HN30 and PCI37A cells were sensitive to cetuximab after HER3 knockdown and NRG1 stimulation (Fig. 4B). This result further confirms that HER3 activation is necessary for cetuximab resistance.

**Fig. 5:**
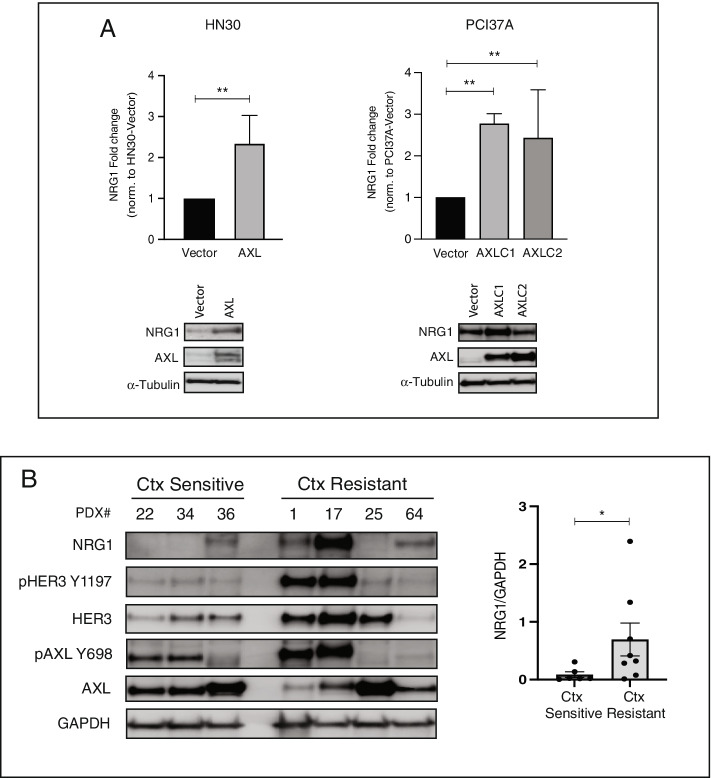
AXL regulates NRG1. **A** The expression levels of NRG1 in HN30-Vector, HN30-AXL PCI37A-Vector and PCI37A-AXL cells were determined by qPCR and immunoblot analysis. α-Tubulin was used as a loading control. Mean values, SEs, and statistical analyses are representative of three independent experiments. *N* = 2–4. **B** Whole cell lysates were harvested from HNSCC PDX tumors and fractionated via SDS-PAGE, followed by an immunoblot for indicated proteins. GAPDH was used as a loading control. The densitometry values represent the mean ratios of NRG1/GAPDH from two independent experiments. Error bars: SEM (*P < 0.05, Mann–Whitney test)

On the basis of these results, we next evaluated whether AXL stimulates NRG1 to regulate cell proliferation in cetuximab-resistant cell lines. Quantitative PCR (qPCR) and immunoblot analysis were used to analyze NRG1 expression levels in HN30-AXL or PCI37A-AXL cells compared to the HN30-vector or PCI37A-vector control, respectively (Fig. 5A, Supplemental Fig. 2). The abundance of NRG1 was increased at both the mRNA and protein levels in HN30-AXL and PCI37A-AXL cells.

To expand these findings, HNSCC patient-derived xenografts (PDXs) were evaluated for NRG1 expression levels (Fig. 5B). Seven HNSCC PDXs were previously characterized and evaluated for cetuximab response [40]. PDX samples were harvested from early-passage tumors and evaluated for NRG1 expression by immunoblot analysis. There were three cetuximab-sensitive PDXs (UWSCC-22, UWSCC-34 and UWSCC-36) and four cetuximab-resistant PDXs (UWSCC-1, UWSCC-17, UWSCC-25 and UWSCC-64). In this small cohort, immunoblot analysis showed that the cetuximab-resistant PDXs on average expressed approximately eight-fold more NRG1 than cetuximab-sensitive PDXs (Fig. 5B, *p < 0.05). Collectively, these data demonstrate that NRG1 is overexpressed in cetuximab-resistant HNSCC.

### AXL regulates NRG1 to lead to cetuximab resistance

**Fig. 6:**
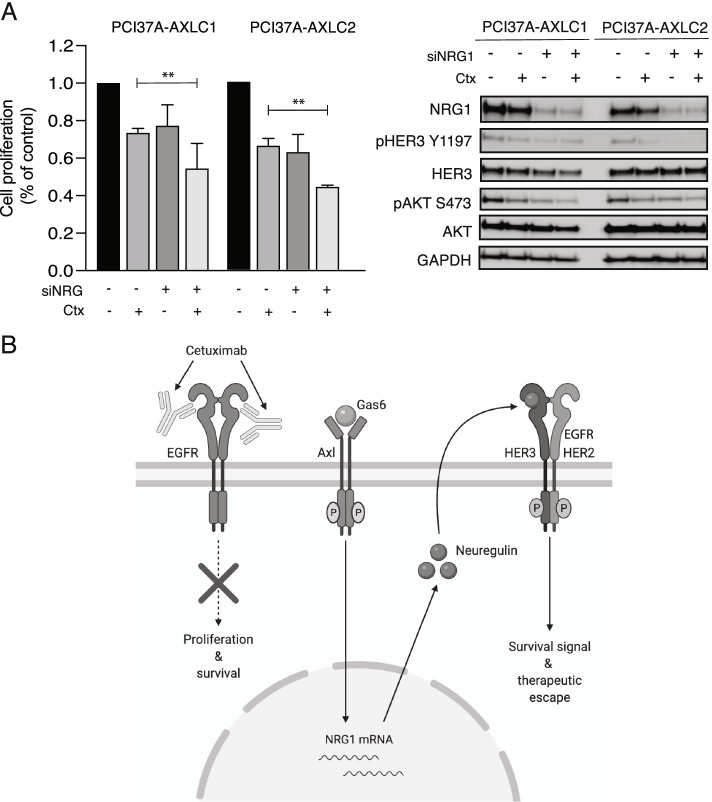
AXL regulates NRG1 to lead to cetuximab resistance. **A** PCI37A-AXL cells were plated and treated with 30 nM of NRG1 siRNA or 30 nM siNT. The next day, cells were treated with 100 nM of 100 nM of IgG or 100 nM cetuximab (Ctx) for 72 h. Cell proliferation was measured after drug treatment using the CCK8 assay. Mean values, SEs, and statistical analyses are representative of seven independent experiments. *N* = 6–10, **P < 0.01. Whole cell lysates were collected at 24 h after treatment, fractioned by SDS-PAGE, and immunoblotted for the indicated proteins. GAPDH was used as a loading control. **B** Proposed model for AXL regulation of NRG1 leading to Ctx resistance

Our data indicated that AXL increased HER3 activation, leading to cetuximab resistance. Furthermore, overexpression of AXL increased NRG1 expression levels in cetuximab-resistant cells, and cetuximab-resistant PDXs have more NRG1 expression than cetuximab-sensitive PDXs (Fig. 5). Thus, we hypothesized that NRG1 may be critical for AXL to mediate cetuximab resistance. To test this, we targeted NRG1 by siRNA in PCI37A-AXL cells and treated with cetuximab for 72 h. Cell proliferation assays indicated that loss of NRG1 expression resensitized cells to cetuximab (Fig. 6A). In PCI37A-AXL cells treated with siNRG1 and cetuximab, there was a substantial decrease in phosphorylation of HER3 and AKT. Collectively, these results demonstrated that AXL is signaling through NRG1 to promote cetuximab resistance.

## Discussion

The current report presents data suggesting that AXL can signal through HER3 via NRG1 to promote cetuximab resistance in HNC. Notably, our models demonstrated that NRG1 is necessary and sufficient for cetuximab resistance. Further investigation revealed that AXL can regulate the expression of NRG1, thereby promoting resistance to cetuximab. Together these findings suggest that AXL and NRG1 expression could predict patient responses to cetuximab therapy and strengthen the rationale for the use of AXL or NRG1 targeted therapies in HNC treatment strategies.

Previous research by our laboratory and other investigators has demonstrated an increase in expression levels of several receptor tyrosine kinases, including AXL and HER3, after development of resistance to EGFR-targeting [32, 41, 42]. Altered expression of AXL in cancer has been studied as a mechanism of acquired resistance to cetuximab [19, 35] and targeting of AXL has been shown to resensitize cells and tumors to EGFR-targeted therapy [22, 36]. Increased expression of EGFR family member HER3 has also been observed in cetuximab-resistant models leading to development of several EGFR-HER3 co-targeting therapeutic strategies [30, 31, 43–45]. In this study, we found that overexpression of AXL leads to cetuximab resistance and increased HER3 activity (Fig. 1), and that HER3 was necessary for AXL to mediate cetuximab resistance (Fig. 2). We also found that HER3 overexpression alone was insufficient for cetuximab resistance in HNSCC cells (Fig. 3). Immunoblot analysis showed that overexpression of HER3 did not increase AXL activity (Fig. 3**)**. These results suggested that overexpression of both AXL and HER3 may be necessary for cetuximab resistance. Despite wide pre-clinical success of HER3 targeting to overcome cetuximab resistance [33, 46, 47], the combination of a HER3 inhibitor with cetuximab did not demonstrate clinical success over cetuximab therapy alone [48–50]. Thus, our lab has continued to investigate the signaling role of HER3 discovering that perhaps AXL and NRG1 are of more importance in the cetuximab resistance pathway.

In the current study, we found that the addition of NRG1 to the cetuximab-sensitive HN30 and PCI37A cell lines rendered these cells resistant to cetuximab (Fig. 4A). In line with this data, we previously reported that NRG1 autocrine signaling is a major driver of acquired resistance to cetuximab [31]. We also found that cetuximab-resistant HN30-AXL and PCI37A-AXLC1 cells relatively expressed more NRG1 than the vector control (Fig. 5A) and that cetuximab-resistant PDX tumors have more NRG1 than cetuximab-sensitive PDXs (Fig. 5B, *p < 0.05). These findings suggested that AXL could regulate NRG1 in cetuximab-resistant HNSCC cells. In addition, analysis of 89 HPV( +) and 409 HPV(-) primary HNC tumor samples in the TCGA as well as 33 HNC cell lines in Cancer Cell Line Encyclopedia (CCLE) showed a positive correlation between AXL and NRG1 mRNA levels (Supplemental Figure S3). Interestingly, PCI37A-AXLC2 cells did not express more NRG1 compared to vector control despite AXL being overexpressed (Fig. 5A), and cetuximab-resistant PDX tumor (UW-SCC25) did not express NRG1 even though they had AXL expression (Fig. 5B). Many groups have begun investigating NRG1 expression in cancer and how this correlates with therapeutic response with varied results. Meetze et al*.* found a significant correlation between NRG1 expression and tumor growth inhibition by the HER3 inhibitory antibody AV-203 [51], and another study demonstrated that HER3 inhibition could be quite effective in NRG1*-*rearranged cancers [52]. Baro et al. also identified upregulation of autocrine NRG1 signaling as a mechanism of cetuximab resistance in HNSCC tumors. Using the HER3 antibody CDX-3379, they were able to overcome cetuximab resistance and enhance tumor growth delay and radiosensitivity [53]. In contrast, one study found that expression of HER3 did not indicate sensitivity to a HER3 antibody or cetuximab. They demonstrated that NRG1 expression along with other EGFR-activation biomarkers correlated with better anti-HER3 response [54]. In this study, knockdown of NRG1 expression by siRNA combined with cetuximab treatment led to diminished cell proliferation by impairing AKT survival signaling in cetuximab-resistant PCI37A-AXL cells (Fig. 6A). Because of the varying results in different cancer models, more investigation of NRG1 expression and cetuximab response must be completed. Additional testing of NRG1 inhibition and resensitization to cetuximab could also be investigated using two high-affinity monoclonal antibodies to NRG1 [55] or an anti-NRG1 antibody that has shown inhibition of tumor growth in preclinical models of pancreatic cancer [56]. Collectively, the data presented within explores a signaling connection between AXL, NRG1, and HER3 in the context of cetuximab resistance in HNC (Fig. 6B).

## Conclusions

We have shown that NRG1 expression, more than HER3 expression, is necessary and sufficient for resistance to cetuximab in our models and that the RTK AXL can regulate expression of NRG1. This data corroborates findings that a combination of HER3 and AXL therapy might be more effective than targeting either alone [57]. Further exploration must be done to identify if the co-expression of AXL and NRG1 could be as used as biomarkers to predict resistance to cetuximab or if a targeting strategy for AXL or NRG1 would provide a therapeutic advantage in the setting of resistance.

## Supplementary Information

**Additional file 1.** Supplemental materials and methods.

**Additional file 2:** **Figure 1.** AXL leads to cetuximab resistance and increased HER3 activity. **Figure 2.** HER3 is necessary for AXL to mediate cetuximab resistance. **Figure 3.** HER3 overexpression alone is insufficient for cetuximab resistance. **Figure 4.** Exogenous expression of NRG1 leads to cetuximab resistance. **Figure 5.** AXL regulates NRG1. **Figure 6.** AXL regulates NRG1 to lead to cetuximab resistance. 

**Additional file 3:** **Supplemental Figure S1.** Endogenous protein expression levels of HN30 and PCI37A cells. **Supplemental Figure S2.** AXL mRNA expression in HN30-AXL and PCI37A-AXLC1 and -AXLC2 cells. **Suppemental Figure S3.** Correlation between AXL and NRG1 mRNA expression levels in TCGA HNC primary tumor samples (A) and CCLE HNC cell lines (B).

## Data Availability

The datasets used and/or analyzed during the current study are available from the corresponding author on reasonable request.

## Abbreviations

CCK8: Cell Counting Kit-8
Ctx: Cetuximab
EGFR: Epidermal Growth Factor Receptor
HNC: Head and Neck cancer
HNSCC: Head and Neck Squamous Cell Carcinomas
NRG1: Neuregulin1
PDXs: Patient-Derived Xenografts
qPCR: Quantitative PCR
RTK: Receptor Tyrosine Kinase
siRNA: Small interfering RNA
siNT: SiNontarget

## References

[1] Bray F, Ferlay J, Soerjomataram I, Siegel RL, Torre LA, Jemal A (2018). Global cancer statistics 2018: GLOBOCAN estimates of incidence and mortality worldwide for 36 cancers in 185 countries. CA Cancer J Clin.

[2] Siegel RL, Miller KD, Jemal A (2020). Cancer statistics, 2020. CA Cancer J Clin.

[3] McDaniel NK, Fischbach SR, Ondracek OJ, Welke NB, Iida M, Wheeler DL. TAM family protein and therapy resistance. In: Kimple RJ, editor. Improving the therapeutic ratio in head and neck cancer. Oxford: Elsevier; 2020. Vol. 6. p. 159–192. ISBN:978-0-12-817868-3.

[4] Pfister DG, Spencer S, Adelstein D, Adkins D, Anzai Y, Brizel DM, Bruce JY, Busse PM, Caudell JJ, Cmelak AJ (2020). Head and Neck Cancers, Version 2.2020, NCCN Clinical Practice Guidelines in Oncology. J Natl Compr Canc Netw.

[5] Specenier P, Vermorken JB (2013). Cetuximab: its unique place in head and neck cancer treatment. Biologics.

[6] Wheeler DL, Dunn EF, Harari PM (2010). Understanding resistance to EGFR inhibitors-impact on future treatment strategies. Nat Rev Clin Oncol.

[7] Kimura H, Sakai K, Arao T, Shimoyama T, Tamura T, Nishio K (2007). Antibody-dependent cellular cytotoxicity of cetuximab against tumor cells with wild-type or mutant epidermal growth factor receptor. Cancer Sci.

[8] Bonner JA, Harari PM, Giralt J, Azarnia N, Shin DM, Cohen RB, Jones CU, Sur R, Raben D, Jassem J (2006). Radiotherapy plus cetuximab for squamous-cell carcinoma of the head and neck. N Engl J Med.

[9] Vermorken JB, Trigo J, Hitt R, Koralewski P, Diaz-Rubio E, Rolland F, Knecht R, Amellal N, Schueler A, Baselga J (2007). Open-label, uncontrolled, multicenter phase II study to evaluate the efficacy and toxicity of cetuximab as a single agent in patients with recurrent and/or metastatic squamous cell carcinoma of the head and neck who failed to respond to platinum-based therapy. J Clin Oncol.

[10] Cai WQ, Zeng LS, Wang LF, Wang YY, Cheng JT, Zhang Y, Han ZW, Zhou Y, Huang SL, Wang XW (2020). The Latest Battles Between EGFR Monoclonal Antibodies and Resistant Tumor Cells. Front Oncol.

[11] Zhang Z, Lee JC, Lin L, Olivas V, Au V, LaFramboise T, Abdel-Rahman M, Wang X, Levine AD, Rho JK (2012). Activation of the AXL kinase causes resistance to EGFR-targeted therapy in lung cancer. Nat Genet.

[12] Linger RM, Cohen RA, Cummings CT, Sather S, Migdall-Wilson J, Middleton DH, Lu X, Baron AE, Franklin WA, Merrick DT (2013). Mer or Axl receptor tyrosine kinase inhibition promotes apoptosis, blocks growth and enhances chemosensitivity of human non-small cell lung cancer. Oncogene.

[13] Byers LA, Diao L, Wang J, Saintigny P, Girard L, Peyton M, Shen L, Fan Y, Giri U, Tumula PK (2013). An epithelial-mesenchymal transition gene signature predicts resistance to EGFR and PI3K inhibitors and identifies Axl as a therapeutic target for overcoming EGFR inhibitor resistance. Clin Cancer Res.

[14] Zhu C, Wei Y, Wei X (2019). AXL receptor tyrosine kinase as a promising anti-cancer approach: functions, molecular mechanisms and clinical applications. Mol Cancer.

[15] Linger RM, Keating AK, Earp HS, Graham DK (2008). TAM receptor tyrosine kinases: biologic functions, signaling, and potential therapeutic targeting in human cancer. Adv Cancer Res.

[16] Feneyrolles C, Spenlinhauer A, Guiet L, Fauvel B, Dayde-Cazals B, Warnault P, Cheve G, Yasri A (2014). Axl kinase as a key target for oncology: focus on small molecule inhibitors. Mol Cancer Ther.

[17] Giles KM, Kalinowski FC, Candy PA, Epis MR, Zhang PM, Redfern AD, Stuart LM, Goodall GJ, Leedman PJ (2013). Axl mediates acquired resistance of head and neck cancer cells to the epidermal growth factor receptor inhibitor erlotinib. Mol Cancer Ther.

[18] von Massenhausen A, Bragelmann J, Billig H, Thewes B, Queisser A, Vogel W, Kristiansen G, Schrock A, Bootz F, Brossart P (2016). Implication of the Receptor Tyrosine Kinase AXL in Head and Neck Cancer Progression. Int J Mol Sci.

[19] Brand TM, Iida M, Stein AP, Corrigan KL, Braverman CM, Luthar N, Toulany M, Gill PS, Salgia R, Kimple RJ (2014). AXL mediates resistance to cetuximab therapy. Cancer Res.

[20] Ye X, Li Y, Stawicki S, Couto S, Eastham-Anderson J, Kallop D, Weimer R, Wu Y, Pei L (2010). An anti-Axl monoclonal antibody attenuates xenograft tumor growth and enhances the effect of multiple anticancer therapies. Oncogene.

[21] Rho JK, Choi YJ, Kim SY, Kim TW, Choi EK, Yoon SJ, Park BM, Park E, Bae JH, Choi CM (2014). MET and AXL inhibitor NPS-1034 exerts efficacy against lung cancer cells resistant to EGFR kinase inhibitors because of MET or AXL activation. Cancer Res.

[22] Taniguchi H, Yamada T, Wang R, Tanimura K, Adachi Y, Nishiyama A, Tanimoto A, Takeuchi S, Araujo LH, Boroni M (2019). AXL confers intrinsic resistance to osimertinib and advances the emergence of tolerant cells. Nat Commun.

[23] Cardone C, Blauensteiner B, Moreno-Viedma V, Martini G, Simeon V, Vitiello PP, Ciardiello D, Belli V, Matrone N, Troiani T (2020). AXL is a predictor of poor survival and of resistance to anti-EGFR therapy in RAS wild-type metastatic colorectal cancer. Eur J Cancer.

[24] Namba K, Shien K, Takahashi Y, Torigoe H, Sato H, Yoshioka T, Takeda T, Kurihara E, Ogoshi Y, Yamamoto H (2019). Activation of AXL as a Preclinical Acquired Resistance Mechanism Against Osimertinib Treatment in EGFR-Mutant Non-Small Cell Lung Cancer Cells. Mol Cancer Res.

[25] Haikala HM, Janne PA (2021). Thirty Years of HER3: From Basic Biology to Therapeutic Interventions. Clin Cancer Res.

[26] Zhang J, Saba NF, Chen GZ, Shin DM (2015). Targeting HER (ERBB) signaling in head and neck cancer: An essential update. Mol Aspects Med.

[27] Pollock NI, Wang L, Wallweber G, Gooding WE, Huang W, Chenna A, Winslow J, Sen M, DeGrave KA, Li H (2015). Increased Expression of HER2, HER3, and HER2:HER3 Heterodimers in HPV-Positive HNSCC Using a Novel Proximity-Based Assay: Implications for Targeted Therapies. Clin Cancer Res.

[28] Rysman B, Mouawad F, Gros A, Lansiaux A, Chevalier D, Meignan S (2016). Human epidermal growth factor receptor 3 in head and neck squamous cell carcinomas. Head Neck.

[29] Takikita M, Xie R, Chung JY, Cho H, Ylaya K, Hong SM, Moskaluk CA, Hewitt SM (2011). Membranous expression of Her3 is associated with a decreased survival in head and neck squamous cell carcinoma. J Transl Med.

[30] Iida M, Bahrar H, Brand TM, Pearson HE, Coan JP, Orbuch RA, Flanigan BG, Swick AD, Prabakaran PJ, Lantto J (2016). Targeting the HER Family with Pan-HER Effectively Overcomes Resistance to Cetuximab. Mol Cancer Ther.

[31] Iida M, Brand TM, Starr MM, Huppert EJ, Luthar N, Bahrar H, Coan JP, Pearson HE, Salgia R, Wheeler DL (2014). Overcoming acquired resistance to cetuximab by dual targeting HER family receptors with antibody-based therapy. Mol Cancer.

[32] Wheeler DL, Huang S, Kruser TJ, Nechrebecki MM, Armstrong EA, Benavente S, Gondi V, Hsu KT, Harari PM (2008). Mechanisms of acquired resistance to cetuximab: role of HER (ErbB) family members. Oncogene.

[33] Wang D, Qian G, Zhang H, Magliocca KR, Nannapaneni S, Amin AR, Rossi M, Patel M, El-Deiry M, Wadsworth JT (2017). HER3 Targeting Sensitizes HNSCC to Cetuximab by Reducing HER3 Activity and HER2/HER3 Dimerization: Evidence from Cell Line and Patient-Derived Xenograft Models. Clin Cancer Res.

[34] Liu X, Liu S, Lyu H, Riker AI, Zhang Y, Liu B (2019). Development of Effective Therapeutics Targeting HER3 for Cancer Treatment. Biol Proced Online.

[35] Brand TM, Iida M, Stein AP, Corrigan KL, Braverman CM, Coan JP, Pearson HE, Bahrar H, Fowler TL, Bednarz BP (2015). AXL Is a Logical Molecular Target in Head and Neck Squamous Cell Carcinoma. Clin Cancer Res.

[36] McDaniel NK, Iida M, Nickel KP, Longhurst CA, Fischbach SR, Rodems TS, Kranjac CA, Bo AY, Luo Q, Gallagher MM (2020). AXL Mediates Cetuximab and Radiation Resistance Through Tyrosine 821 and the c-ABL Kinase Pathway in Head and Neck Cancer. Clin Cancer Res.

[37] McDaniel NK, Cummings CT, Iida M, Hulse J, Pearson HE, Vasileiadi E, Parker RE, Orbuch RA, Ondracek OJ, Welke NB (2018). MERTK Mediates Intrinsic and Adaptive Resistance to AXL-targeting Agents. Mol Cancer Ther.

[38] Swick AD, Prabakaran PJ, Miller MC, Javaid AM, Fisher MM, Sampene E, Ong IM, Hu R, Iida M, Nickel KP (2017). Cotargeting mTORC and EGFR Signaling as a Therapeutic Strategy in HNSCC. Mol Cancer Ther.

[39] Li C, Brand TM, Iida M, Huang S, Armstrong EA, van der Kogel A, Wheeler DL (2013). Human epidermal growth factor receptor 3 (HER3) blockade with U3–1287/AMG888 enhances the efficacy of radiation therapy in lung and head and neck carcinoma. Discov Med.

[40] Kimple RJ, Harari PM, Torres AD, Yang RZ, Soriano BJ, Yu M, Armstrong EA, Blitzer GC, Smith MA, Lorenz LD (2013). Development and characterization of HPV-positive and HPV-negative head and neck squamous cell carcinoma tumorgrafts. Clin Cancer Res.

[41] Leonard B, Brand TM, O'Keefe RA, Lee ED, Zeng Y, Kemmer JD, Li H, Grandis JR, Bhola NE (2018). BET Inhibition Overcomes Receptor Tyrosine Kinase-Mediated Cetuximab Resistance in HNSCC. Cancer Res.

[42] Hu S, Dai H, Li T, Tang Y, Fu W, Yuan Q, Wang F, Lv G, Lv Y, Fan X (2016). Broad RTK-targeted therapy overcomes molecular heterogeneity-driven resistance to cetuximab via vectored immunoprophylaxis in colorectal cancer. Cancer Lett.

[43] Rau A, Lieb WS, Seifert O, Honer J, Birnstock D, Richter F, Aschmoneit N, Olayioye MA, Kontermann RE (2020). Inhibition of Tumor Cell Growth and Cancer Stem Cell Expansion by a Bispecific Antibody Targeting EGFR and HER3. Mol Cancer Ther.

[44] Lee S, Greenlee EB, Amick JR, Ligon GF, Lillquist JS, Natoli EJ, Hadari Y, Alvarado D, Schlessinger J (2015). Inhibition of ErbB3 by a monoclonal antibody that locks the extracellular domain in an inactive configuration. Proc Natl Acad Sci U S A.

[45] Francis DM, Huang S, Armstrong EA, Werner LR, Hullett CR, Li C (2015). Pan-HER receptor inhibition augments radiation response in human lung and head and neck cancer models. Clin Cancer Res..

[46] Huang S, Li C, Armstrong EA, Peet CR, Saker J, Amler LC, Sliwkowski MX, Harari PM (2013). Dual targeting of EGFR and HER3 with MEHD7945A overcomes acquired resistance to EGFR inhibitors and radiation. Cancer Res.

[47] Kawakami H, Okamoto I, Yonesaka K, Okamoto K, Shibata K, Shinkai Y, Sakamoto H, Kitano M, Tamura T, Nishio K (2014). The anti-HER3 antibody patritumab abrogates cetuximab resistance mediated by heregulin in colorectal cancer cells. Oncotarget.

[48] Fayette J, Wirth L, Oprean C, Udrea A, Jimeno A, Rischin D, Nutting C, Harari PM, Csoszi T, Cernea D (2016). Randomized Phase II Study of Duligotuzumab (MEHD7945A) vs. Cetuximab in Squamous Cell Carcinoma of the Head and Neck (MEHGAN Study). Front Oncol.

[49] Forster MD, Dillon MT, Kocsis J, Remenar E, Pajkos G, Rolland F, Greenberg J, Harrington KJ (2019). Patritumab or placebo, with cetuximab plus platinum therapy in recurrent or metastatic squamous cell carcinoma of the head and neck: A randomised phase II study. Eur J Cancer.

[50] Meulendijks D, Jacob W, Voest EE, Mau-Sorensen M, Martinez-Garcia M, Taus A, Fleitas T, Cervantes A, Lolkema MP, Langenberg MHG (2017). Phase Ib Study of Lumretuzumab Plus Cetuximab or Erlotinib in Solid Tumor Patients and Evaluation of HER3 and Heregulin as Potential Biomarkers of Clinical Activity. Clin Cancer Res.

[51] Meetze K, Vincent S, Tyler S, Mazsa EK, Delpero AR, Bottega S, McIntosh D, Nicoletti R, Winston WM, Weiler S (2015). Neuregulin 1 expression is a predictive biomarker for response to AV-203, an ERBB3 inhibitory antibody, in human tumor models. Clin Cancer Res.

[52] Drilon A, Somwar R, Mangatt BP, Edgren H, Desmeules P, Ruusulehto A, Smith RS, Delasos L, Vojnic M, Plodkowski AJ (2018). Response to ERBB3-Directed Targeted Therapy in NRG1-Rearranged Cancers. Cancer Discov.

[53] Baro M, Lopez Sambrooks C, Burtness BA, Lemmon MA, Contessa JN (2019). Neuregulin Signaling Is a Mechanism of Therapeutic Resistance in Head and Neck Squamous Cell Carcinoma. Mol Cancer Ther.

[54] Alvarado D, Ligon GF, Lillquist JS, Seibel SB, Wallweber G, Neumeister VM, Rimm DL, McMahon G, LaVallee TM (2017). ErbB activation signatures as potential biomarkers for anti-ErbB3 treatment in HNSCC. PLoS One.

[55] Hegde GV, de la Cruz CC, Chiu C, Alag N, Schaefer G, Crocker L, Ross S, Goldenberg D, Merchant M, Tien J (2013). Blocking NRG1 and other ligand-mediated Her4 signaling enhances the magnitude and duration of the chemotherapeutic response of non-small cell lung cancer. Sci Transl Med.

[56] Ogier C, Colombo PE, Bousquet C, Canterel-Thouennon L, Sicard P, Garambois V, Thomas G, Gaborit N, Jarlier M, Pirot N (2018). Targeting the NRG1/HER3 pathway in tumor cells and cancer-associated fibroblasts with an anti-neuregulin 1 antibody inhibits tumor growth in pre-clinical models of pancreatic cancer. Cancer Lett.

[57] Torka R, Penzes K, Gusenbauer S, Baumann C, Szabadkai I, Orfi L, Keri G, Ullrich A (2014). Activation of HER3 interferes with antitumor effects of Axl receptor tyrosine kinase inhibitors: suggestion of combination therapy. Neoplasia.

