# Supplementary material for: AXL regulates neuregulin1 expression leading to cetuximab resistance in head and neck cancer
Source: BMC Cancer. 2022 Apr 23;22:447. doi: 10.1186/s12885-022-09511-6 (PMC9035247; doi:10.1186/s12885-022-09511-6)
Supplement: Supplementary file 1 — Additional file 1. Supplemental materials and methods. [file 12885_2022_9511_MOESM1_ESM.pdf]

Supplemental Materials and Methods

Cell Lines. All cell lines were obtained and validated by the following sources.

Cell Line	Sources	Culture Condition
UMSCC1	UW SPORE[3]	DMEM with 4.5 g/dL glucose, 10% FBS, penicillin (100 units/mL), streptomycin (100 mg/mL)
UMSCC6		
HN30	Dr. Ravi Salgia, City of Hope, Duarte, CA, USA	
PCI37A	Dr. Jennifer Grandis, UCSF, CA, USA	

HNSCC PDXs. All PDXs tumors were obtained from UW SPORE resources[3].

UW SCC#[1,2]	Cetuximab[2]	Age	Gender	Tobacco (pack-yrs)	Alcohol	T stage	Differentiation	HPV (p16)
22	Sensitive	57	F	0	Occasional	T4	Well	-
34	Sensitive	69	M	10	Moderate	T1	Moderate	-
36	Sensitive	68	M	7	Occasional	T2	Moderate	+
1	Resistant	57	M	0	Occasional	T2	Moderate	+
17	Resistant	80	M	0	Occasional	T3	Poor	+
25	Resistant	76	M	30	Heavy	T4	Moderate	-
64	Resistant	53	M	20	Moderate	T2	Well	-

HNC primary tumor and cell line samples for analysis. Analysis of correlation between AXL and NRG1 mRNA expression levels was performed using 1) The Cancer Genome Atlas (TCGA) HNSCC dataset which includes patient classification of patient HPV status (https://portal.gdc.cancer.gov/), 2) HNC cell line dataset in The Cancer Cell Line Encyclopedia (CCLE) (https://sites.broadinstitute.org/ccle/).

References

1. Kimple RJ, Harari PM, Torres AD, Yang RZ, Soriano BJ, Yu M, et al. Development and characterization of HPV-positive and HPV-negative head and neck squamous cell carcinoma tumorgrafts. Clin Cancer Res. 2013, 19:855-64.
2. Swick AD, Prabakaran PJ, Miller MC, Javaid AM, Fisher MM, Sampene E, Ong IM, Hu R, Iida M, Nickel KP et al: Cotargeting mTORC and EGFR Signaling as a Therapeutic Strategy in HNSCC. Mol Cancer Ther 2017, 16(7):1257-1268.
3. Wisconsin Head and Neck Cancer SPORE (https://hn-spore.wisc.edu/)
