# Supplementary material for: AXL regulates neuregulin1 expression leading to cetuximab resistance in head and neck cancer
Source: BMC Cancer. 2022 Apr 23;22:447. doi: 10.1186/s12885-022-09511-6 (PMC9035247; doi:10.1186/s12885-022-09511-6)
Supplement: Supplementary file 2 — Additional file 2: Figure 1. AXL leads to cetuximab resistance and increased HER3 activity. Figure 2. HER3 is necessary for AXL to mediate cetuximab resistance. Figure 3. HER3 overexpression alone is insufficient for cetuximab resistance. Figure 4. Exogenous expression of NRG1 leads to cetuximab resistance. Figure 5. AXL regulates NRG1. Figure 6. AXL regulates NRG1 to lead to cetuximab resistance. [file 12885_2022_9511_MOESM2_ESM.pdf]

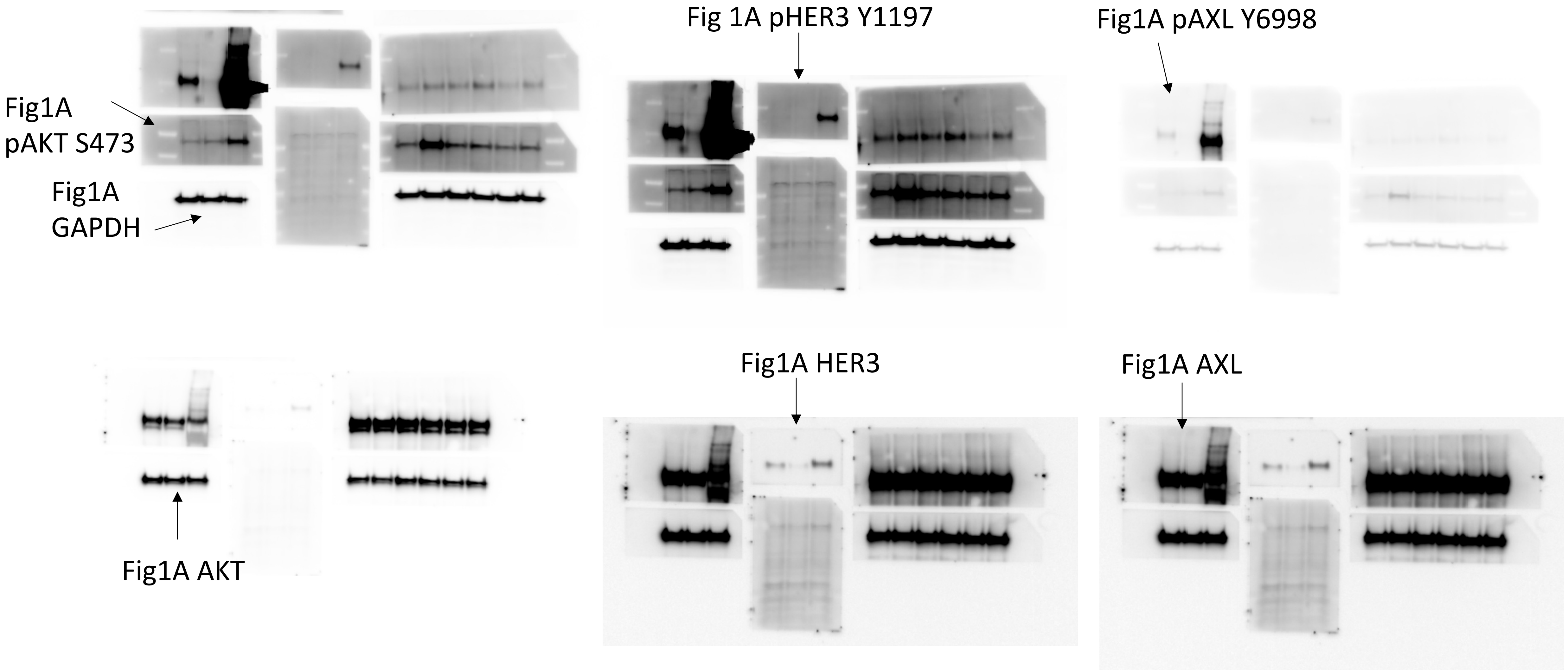

Figure 1: AXL leads to cetuximab resistance and increased HER3 activity.
A: HN30 cells were treated with 100nM of IgG, 100nM of cetuximab (Ctx), or combination of cetuximab and Gas6 (200ng/uL) for 72 hours and relative cell numbers were determined by CCK8 assay. Mean values, SEs, and statistical analyses are representative of two independent experiments. N=3, $^{**}P<0.01$. Whole cell lysates were harvested and fractionated via SDS-PAGE, followed by immunoblotting for the indicated proteins. GAPDH was used as a loading control.

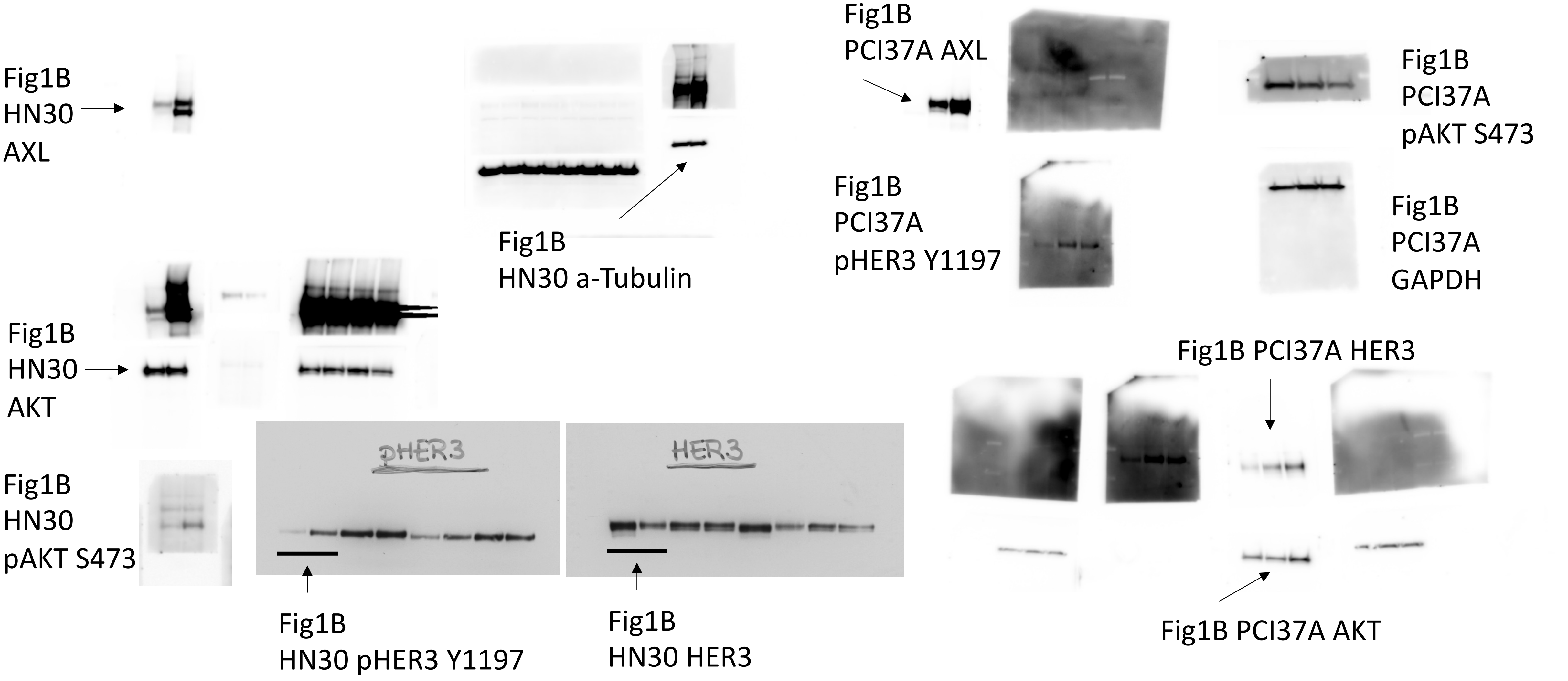

Figure 1: AXL leads to cetuximab resistance and increased HER3 activity.

B: Cell proliferation in AXL overexpressed cells was measured via CCK8 assay after 72 hours of treatment with cetuximab and relative values were determined. Mean values, SEs, and statistical analyses are representative of three independent experiments. N=3, **P<0.01. Whole cell lysates were harvested and fractionated via SDS-PAGE, followed by immunoblotting for the indicated proteins. a-Tubulin and GAPDH were used as loading controls.

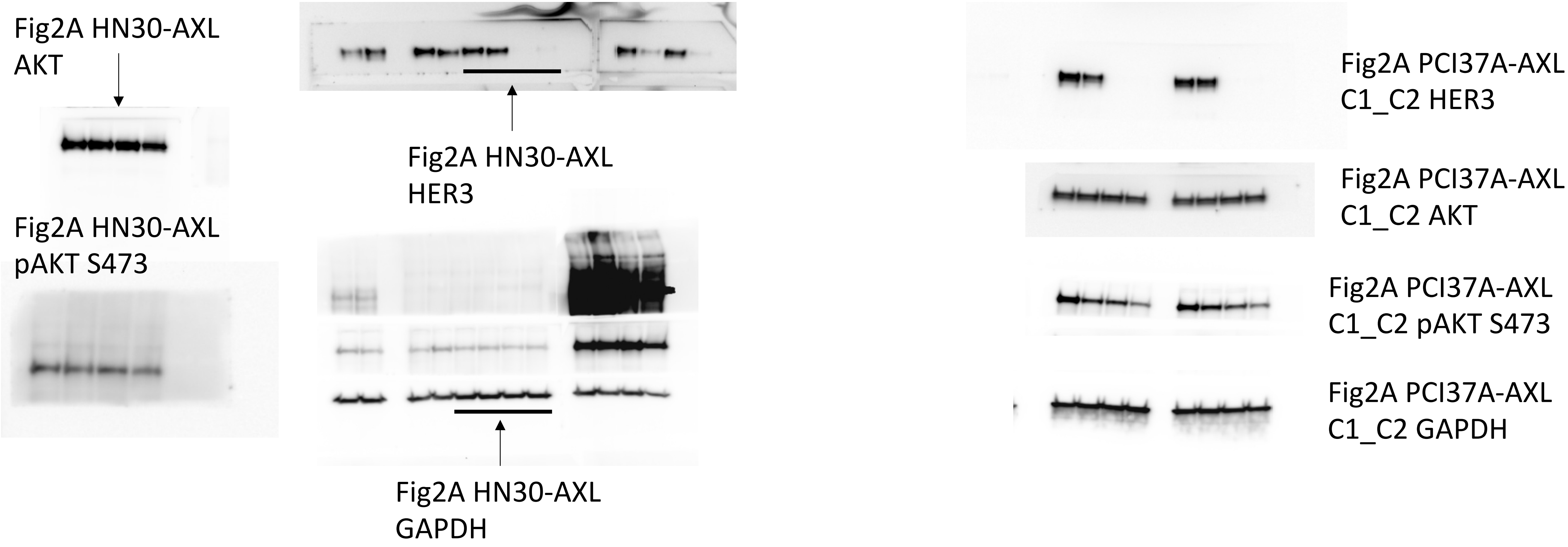

Figure 2: HER3 is necessary for AXL to mediate cetuximab resistance.

A: HN30-AXL and PCI37A-AXL cells were plated and treated with 30nM of HER3 siRNA (siHER3) or 30nM non-target siRNA (siNT). The next day, cells were treated with 100nM of IgG or 100nM cetuximab for 72 hours. Growth was measured after drug treatment by CCK8 assay. Mean values, SEs, and statistical analyses are representative of three independent experiments. N=5-10, ***P*<0.01. Whole cell lysates were collected 24 hours after treatment, fractionated by SDS-PAGE and immunoblotted for the indicated proteins. GAPDH was used as a loading control.

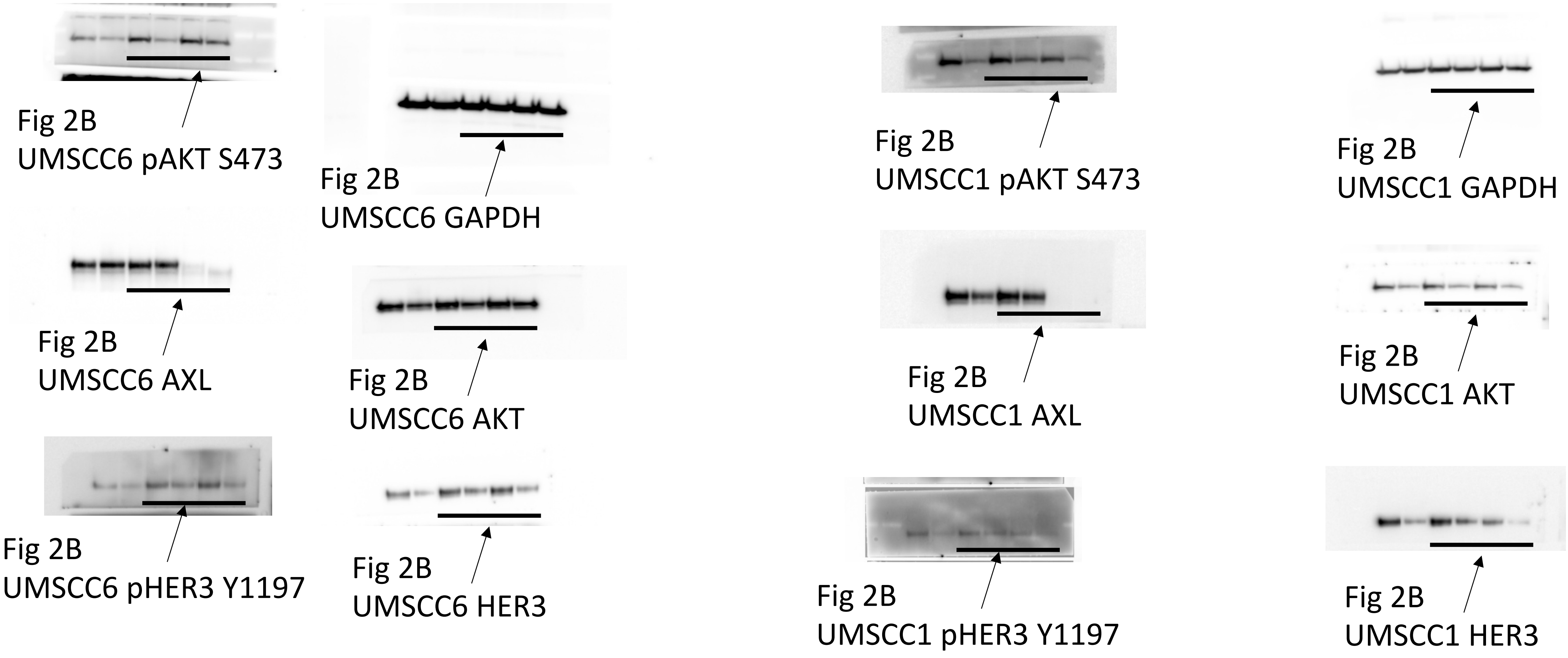

Figure 2: HER3 is necessary for AXL to mediate cetuximab resistance.
B: UMSCC1 and UMSCC6 HNSCC cells were plated and treated with 30nM of siAXL or 30nM non-target siRNA. The next day, cells were treated with 100nM of IgG or 100nM cetuximab for 72 hours. Cell proliferation was measured after drug treatment by CCK8 assay. Mean values, SEs, and statistical analyses are representative of three independent experiments. N=3, **P<0.01. Whole cell lysates were collected at 24 hours after treatment, fractionated by SDS-PAGE, and immunoblotted for the indicated proteins. GAPDH was used as a loading control.

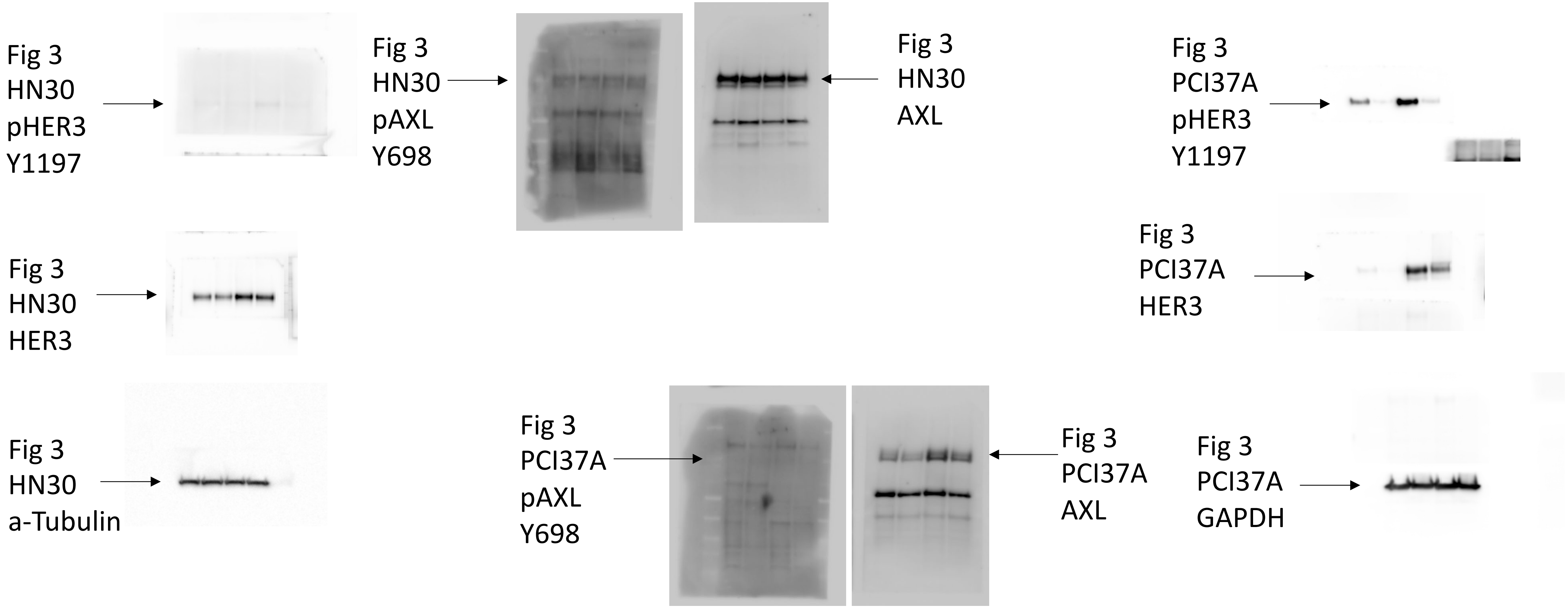

Figure 3: HER3 overexpression alone is insufficient for cetuximab resistance.

HN30 and PCI37A cells stably overexpressing HER3 or the pcDNA6.0 vector were treated with 100nM of cetuximab for 72 hours before performing crystal violet or CCK8 proliferation assays. Whole cell lysate was harvested at 24 hours after treatment and subjected to immunoblot analysis following fractionation via SDS-PAGE. GAPDH or a-Tubulin was used as a loading control. Mean values, SEs, and statistical analyses are representative of two or three independent experiments. N=3-6. NS: not significant.

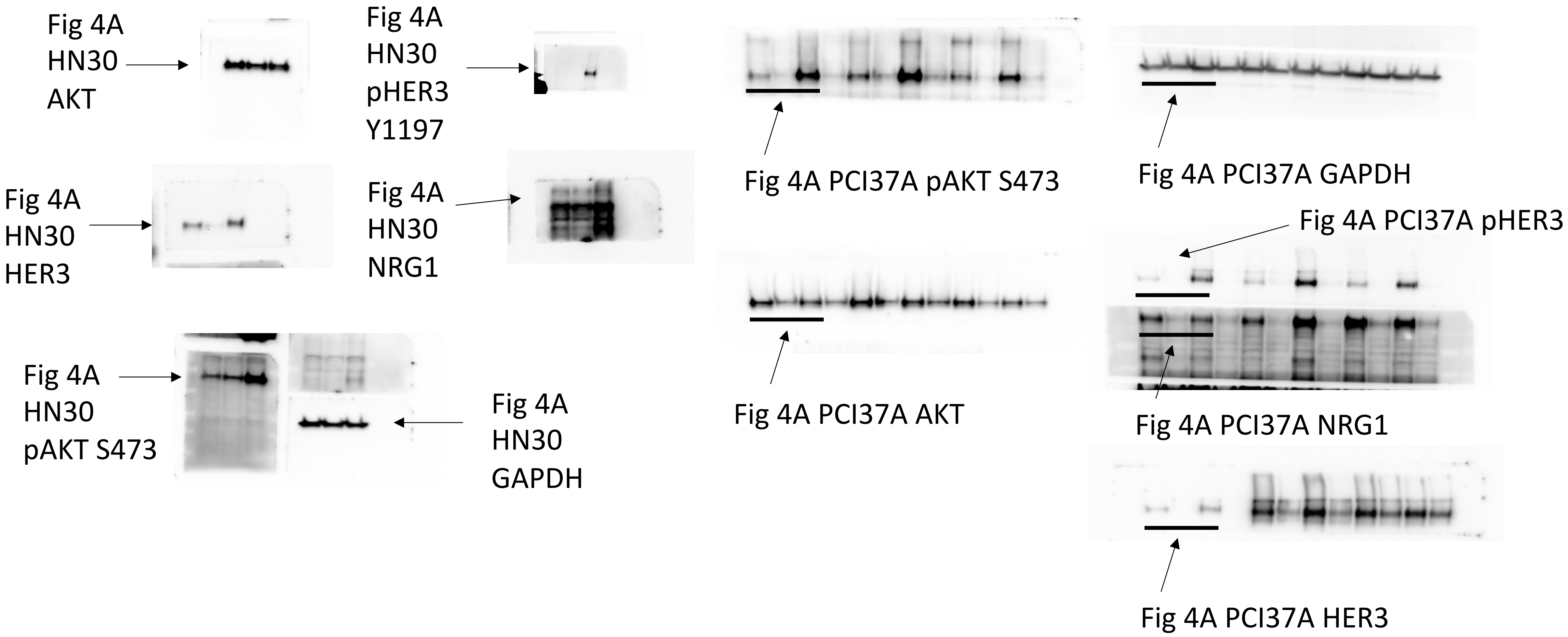

Figure 4: Exogenous expression of NRG1 leads to cetuximab resistance.

A: HN30 and PCI37A cells were plated and treated with 100nM of cetuximab, 100ng/mL of NRG1, or the combination of cetuximab and NRG1 for 72 hours. Relative cell numbers were determined by crystal violet or CCK8 assay. Mean values, SEs, and statistical analyses are representative of two independent experiments. N=6, ***P*<0.01. Whole cell lysates were harvested at 24 hours after treatment and fractionated via SDS-PAGE, followed by immunoblotting for the indicated proteins. GAPDH was used as a loading control.

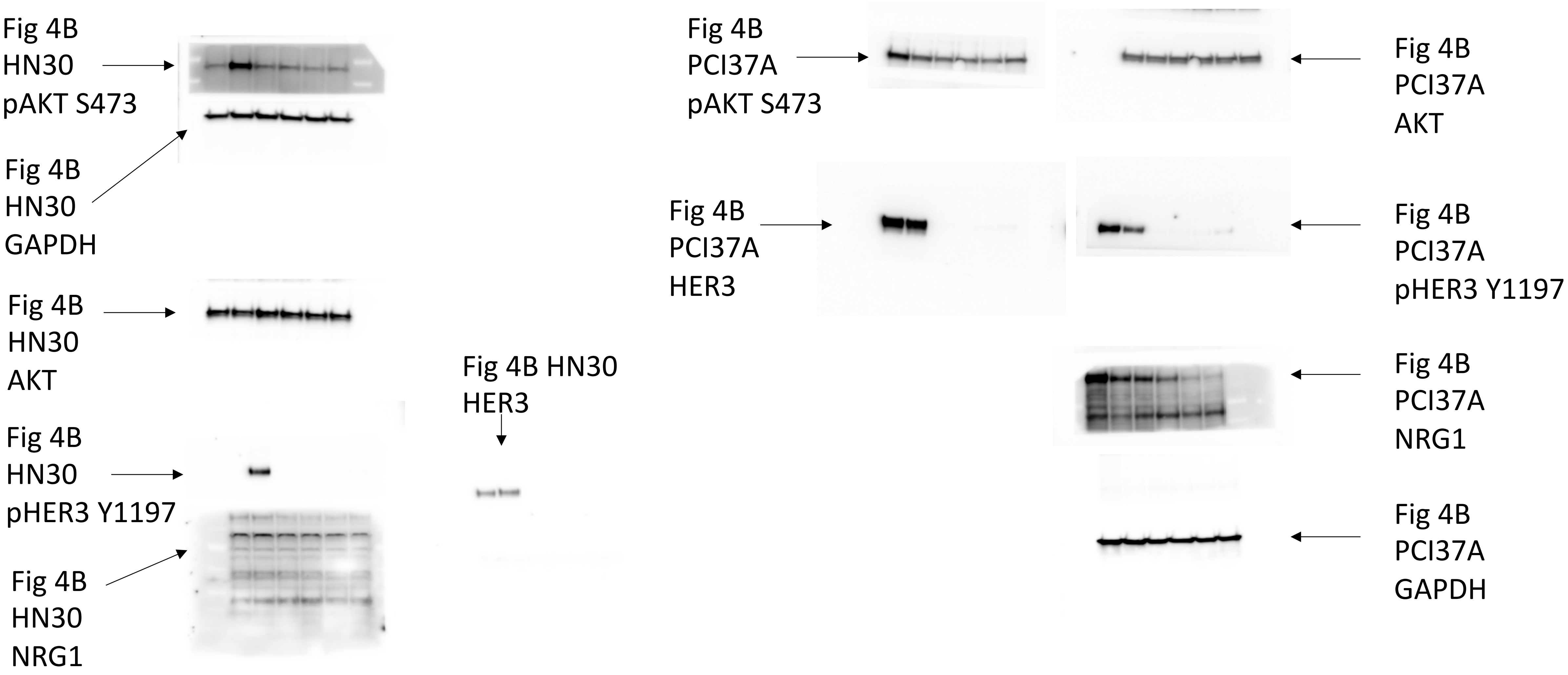

Figure 4: Exogenous expression of NRG1 leads to cetuximab resistance.

B: HN30 and PCI37A cells were transfected with 30 nM siHER3 or 30nM siNT for 24 hours before treatment with cetuximab (100nM) or NRG1 (100 ng/ml) for an additional 72 hours. Relative cell numbers were determined by CCK8 assay. Mean values, SEs, and statistical analyses are representative of two or three independent experiments. N=3-10, ***P*<0.01. Whole cell lysate was harvested at 24 hours after treatment and subjected to immunoblot analysis following fractionation via SDS-PAGE. GAPDH was used as loading.

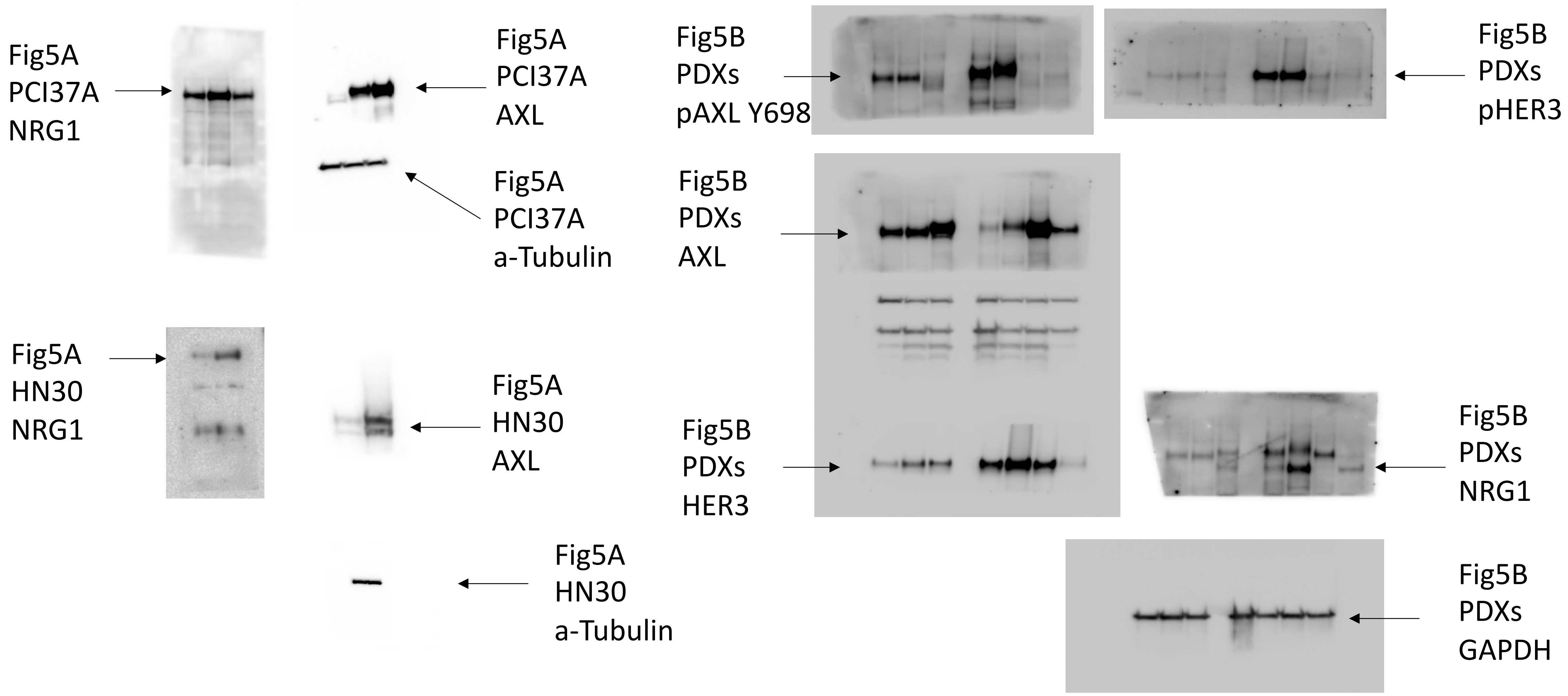

Figure 5: AXL regulates NRG1.

A: The expression levels of NRG1 in HN30-Vector, HN30-AXL PCI37A-Vector and PCI37A-AXL cells were determined by qPCR and immunoblot analysis. a-Tubulin was used as a loading control. Mean values, SEs, and statistical analyses are representative of three independent experiments. N=2-4. B: Whole cell lysates were harvested from HNSCC PDX tumors and fractionated via SDS-PAGE, followed by an immunoblot for NRG1, HER3, and AXL. GAPDH was used as a loading control.

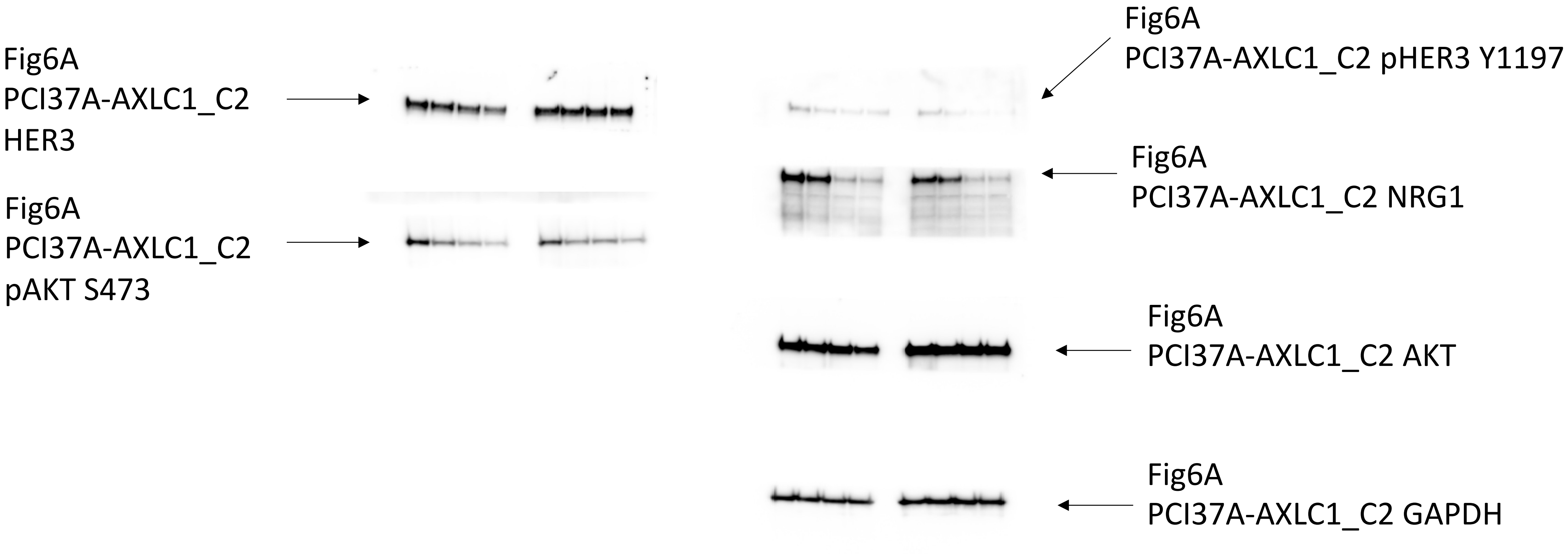

Figure 6: AXL regulates NRG1 to lead to cetuximab resistance.
A: PCI37A-AXL cells were plated and treated with 30nM of NRG1 siRNA or 30nM non-targeting siRNA. The next day, cells were treated with 100nM of 100nM of IgG or 100nM cetuximab for 72 hours. Growth was measured after drug treatment using the CCK8 assay. Mean values, SEs, and statistical analyses are representative of seven independent experiments. N=6-10, **P<0.01. Whole cell lysates were collected at 24 hours after treatment, fractioned by SDS-PAGE, and immunoblotted for the indicated proteins. GAPDH was used as a loading control.

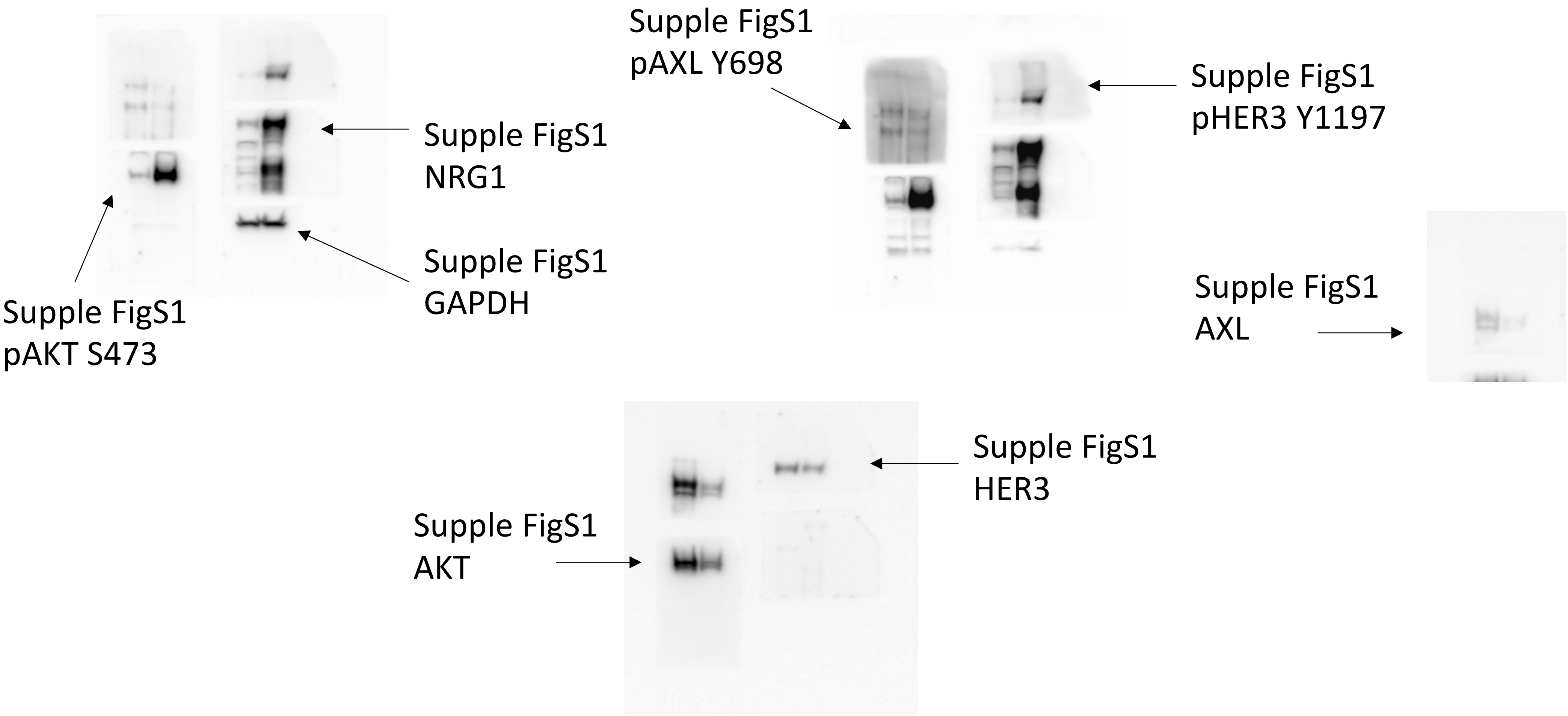

Supplemental Figure S1: Endogenous protein expression level of HN30 and PCI37A cells.
Endogenous protein expression level of HN30 and PCI37Alysates was determined by immunoblot analysis with GAPDH as loading control.
