# Supplementary material for: AXL regulates neuregulin1 expression leading to cetuximab resistance in head and neck cancer
Source: BMC Cancer. 2022 Apr 23;22:447. doi: 10.1186/s12885-022-09511-6 (PMC9035247; doi:10.1186/s12885-022-09511-6)
Supplement: Supplementary file 3 — Additional file 3: Supplemental Figure S1. Endogenous protein expression levels of HN30 and PCI37A cells. Supplemental Figure S2. AXL mRNA expression in HN30-AXL and PCI37A-AXLC1 and -AXLC2 cells. Suppemental Figure S3. Correlation between AXL and NRG1 mRNA expression levels in TCGA HNC primary tumor samples (A) and CCLE HNC cell lines (B). [file 12885_2022_9511_MOESM3_ESM.pdf]

Supplemental Figure S1

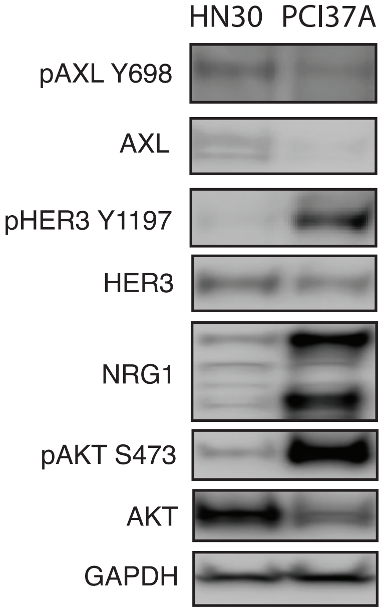

Supplemental Figure S2

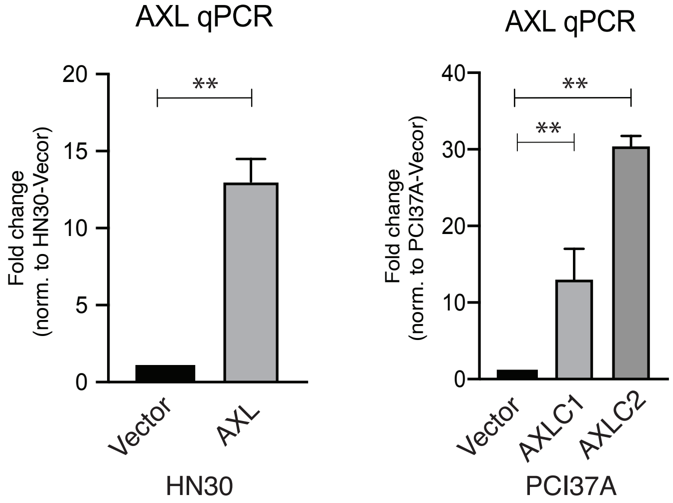

Supplemental Figure S3A

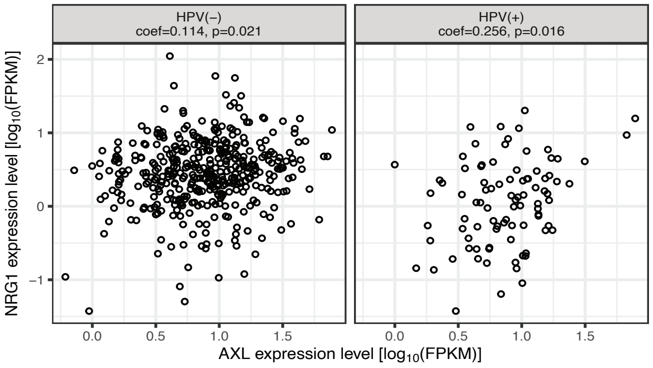

Supplemental Figure S3B

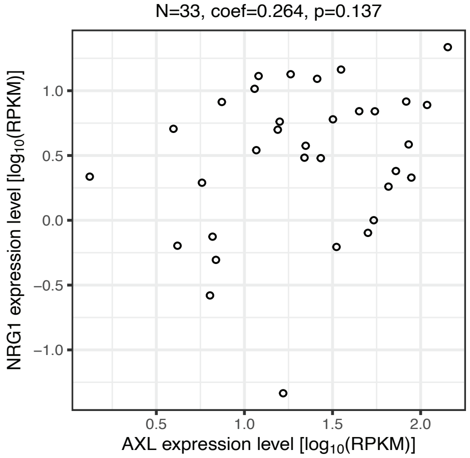

Supplemental Figure S1: Endogenous protein expression levels of HN30 and PCI37A cells. Endogenous protein expression levels of HN30 and PCI37A lysates was determined by immunoblot analysis with GAPDH as a loading control.

Supplemental Figure S2: AXL mRNA expression in HN30-AXL and PCI37A-AXLC1 and -AXLC2 cells. AXL mRNA expression in HN30-AXL and PCI37A-AXLC1 and -AXLC2 cells was detected by qPCR and normalized to AXL expression in their vector cells. (n=3 in three independent experiments).

Suppemental Figure S3: Correlation between AXL and NRG1 mRNA expression levels in TCGA HNC primary tumor samples (A) and CCLE HNC cell lines (B). Each dot represents an individual sample. P-value and correlation coefficient are shown (Spearman Rank correlation).
N=89 HPV(+) and 409 HPV(-) from TCGA, N=33 from from Cancer Cell Line Encyclopedia (CCLE).
